# “It’s Difficult, There’s No Formula”: Qualitative Study of Stroke Related Communication Between Primary and Secondary Healthcare Professionals

**DOI:** 10.5334/ijic.5465

**Published:** 2020-11-10

**Authors:** Maria Raisa Jessica (Ryc) Aquino, Ricky Mullis, Caroline Moore, Elizabeth Kreit, Lisa Lim, Christopher McKevitt, Bundy Mackintosh, Jonathan Mant

**Affiliations:** 1 Primary Care Unit, Department of Public Health and Primary Care, University of Cambridge, GB; 2 Population Health Sciences Institute, Newcastle University, GB; 3 The Open University, GB; 4 Department of Population Health Sciences, Kings College London, GB; 5 Department of Psychology, University of Essex, GB

**Keywords:** primary health care, specialist care, stroke, interprofessional communication, transition of care, integrated care

## Abstract

**Introduction:**

Stroke survivors have complex health needs requiring long-term, integrated care. This study aimed to elicit generalists’ and specialists’ experience of stroke-related interprofessional communication, including perceived barriers and enablers.

**Design and Setting:**

Qualitative study involving generalist (primary care) and specialist services (acute and community) in England. Six focus groups (n = 48) were conducted.

**Method:**

Healthcare professionals were purposively selected and invited to participate. Audio-recordings were transcribed verbatim and analysed using Framework Analysis.

**Results:**

Four themes were identified: 1) Generalists and specialists have overlapping roles but are working in silos; 2) Referral decision-making process as influential to generalist-specialist communication; 3) Variable quality of communication; and 4) Improved dialogue between generalist and specialist services.

**Conclusions:**

Generalists and specialists recognise the need for better communication with each other. Current care is characterised by silo-based working that ignores the contribution of other sectors. Failure to bridge this communication gap will result in people with stroke continuing to experience unmet stroke needs and fragmented care.

## Introduction

The proportion of people living with stroke is growing [1]. In England, National Institute for Health and Care Excellence (NICE) Guidelines for stroke recommend that a person with suspected or confirmed stroke event is admitted to a specialist stroke unit, in order to receive required treatment promptly [2]. Stroke rehabilitation follows, which involves providing stroke survivors with support and treatment from a multidisciplinary rehabilitation team. Stroke survivors’ transition from acute settings to rehabilitation can take place either in hospital, at home or the community. One model of care is Early Supported Discharge (ESD), which offers community-based health and social care as an alternative to inpatient care [2]. NICE recommend that transfers of care from hospital to community should include all pertinent health and social care information, given to relevant health and social care professionals and patients promptly [2]. Long-term care led by generalists in the community is recommended [2,3,4] and stroke survivors are encouraged to self-refer if any issues arise [2]. However, it is unclear whether primary care models of care are effective for addressing stroke survivors’ and carers’ unmet needs [4], and challenges to implementing integrated care remain [5,6]. For instance, ESD was only offered to 34.6% of eligible patients in a 2017 National Audit [7]. Cochrane reviews show that ESD for stroke does reduce hospital lengths of stay [8], but taken as a whole, early discharge services for adults (including stroke) have no effect on mortality [9].

Integrated care models, where distinct but related structures such as health and social care providers and organisations interact [10], are increasingly common [11,12]. However, beyond ESD, integrated care for stroke remains underdeveloped both in the UK and internationally [5,13] and patients and carers have described follow-up care as fragmented [14,6]. Integrated stroke care services require good information exchange between healthcare professionals after discharge, as part of addressing survivors’ and carers’ needs for continued support [15].

Integrated care models typically comprise multidisciplinary teams including community-based services and specialist acute stroke and rehabilitation services working collaboratively [10,11]. Integration of generalist and specialist services are especially challenging as it requires different sectors to agree to not only share particular processes and fiscal arrangements [10], but also values and goals amongst a large group of practitioners from discrete disciplinary backgrounds [4]. Stroke is a good exemplar condition in which to explore these issues since many stroke survivors have ongoing physical and mental health needs (e.g. speech, mobility, and emotional difficulties) [14,16] which require input from stroke specialists and generalist clinicians [17]. Since around half of stroke survivors report unmet needs up to five years poststroke [3], it is likely that current integration is not optimal.

Stroke survivors and caregivers report poor communication and continuity of care between healthcare services, and insufficient follow-up from healthcare professionals [14]. Healthcare providers associate this with misunderstanding of each other’s roles and time pressures [18]. Similarly, patients report having unmet needs and feeling unsupported in the long-term [14,16,19]. Good communication is characteristic of high-quality care transfers [14,20,21], but the extent to which timely and accurate information transfer occurs from hospital to community for stroke survivors remains unclear [12]. Methods of generalist-specialist communication concerning stroke management varies [20] and includes phone calls, case conferences, and care planning meetings [19]. Our understanding of communication processes and quality between generalists and specialists during transition from hospital to home remains scarce [18,20]. To our knowledge, no study of generalist and specialist healthcare providers’ communication concerning stroke care following hospital discharge exists.

As part of a larger research programme to develop a new model of primary care for stroke survivors living in the community [22], we aimed to explore generalist-specialist communication concerning long-term stroke care following hospital discharge, specifically: (1) What are the communication processes between generalists and specialists concerning stroke care after hospital discharge? (2) What are the barriers and enablers to communication between these groups?

## Method

### Design and participants

Focus groups were conducted with generalists and specialist healthcare professionals delivering stroke care. Participants were recruited via six National Health Service (NHS) acute Trusts (East of England and East Midlands) and Clinical Research Networks in these regions. Invitation packs containing the study invitation, participant information sheet, reply slip, consent form, and focus group materials were sent to 91 individuals by email. Purposive combined with snowball sampling ensured a broad range of experience, knowledge and perspectives reflecting real-life practice were included. Written consent was obtained from participants prior to participation in the focus groups. Participants were reimbursed for their time (healthcare professionals’ respective hourly rates) and travel expenses.

### Data collection and analysis

Six focus groups were conducted in 2016 with experienced facilitators (2 per group; DP, CM, RM), lasting 68 minutes on average (range = 67–85 min). Focus groups encouraged the exchange of multiple views and experiences and provided participants opportunities for instantaneous clarification and re-evaluation of individual perspectives [23]. Gathering these professionals together allows for better understanding of interprofessional communication processes and opportunities to develop potential solutions to overcoming communication barriers as healthcare providers with discrete but overlapping roles. A topic guide (Supporting Text 1) was developed with input from stroke patient groups, concentrating on: 1) roles of generalists and specialists in post-stroke care, 2) re-referrals to services, 3) guidelines relating to stroke rehabilitation, and 4) communication between specialists and generalists. Participants also undertook two group-based exercises to elicit further discussion [23]: 1) using clinical vignettes (e.g. expressive aphasia, fatigue, cognition, motor function, emotional problems) to explore indications for referral and 2) implementation of clinical guidelines [7,24] in practice. Data were analysed iteratively, using Framework Analysis [25]. Two researchers (DP, RA) undertook the analysis, starting with the familiarisation phase. Initial coding was done independently. Then thematic framework was developed using coded data from two transcripts and then discussed to form a consensus on the framework going forward (with input from RM). The data were then charted, and summarised, and sub-themes were generated and refined. Finally, data were mapped and interpreted, through comparing data summaries with the originally charted data and identifying relationships between these. Themes identified were verified through discussion with focus group moderators (RM, CM), which helped to establish trustworthiness in the data. All focus groups were audio-recorded, professionally transcribed verbatim, and data managed using NVivo V.11.

## Results

Of 66 healthcare professionals expressing interest, 48 (79% female) consented to and participated in the study. Participants were from various geographical settings, where there were differences in how services were provided. Most had over 5 years’ experience, with only 5 (10%) participants having 1–5 years’ experience. Each group had 6–12 participants. Group characteristics are summarised in Table 1.

**Table 1:** Group-level characteristics.

Group ID	Setting; population size	Group composition		Group size (N = 48)

		Specialists	Generalists	

Site 01	City, East of England; population <150,000	Acute care speech and language therapist = 1 Acute care occupational therapist = 1 Stroke consultant = 1 Community occupational therapist = 1 Community speech and language therapist = 1	GP = 1 Nurse = 1	7
Site 02	City, East of England; population >200,000	Acute care stroke nurse = 1 Acute care speech and language therapist = 1 Acute care occupational therapist = 1 Community occupational therapist = 1	GP = 2 Nurse = 1	7
Site 03	Town, East of England; population of <200,000	Acute care occupational therapist = 1 Acute care physiotherapist = 1 Community physiotherapist = 1 Assistant practitioner = 1	GP = 1 Nurse = 1	6
Site 04	City, East Midlands population >300,000	Acute care occupational therapist = 1 Acute care physiotherapist = 1 Acute care nurse = 1 Clinical specialist for stroke = 1 Stroke review officer = 1 Community physiotherapist = 1	GP = 2 Nurse = 1	9
Site 05	City, East Midlands; population <300,000	Acute care nurse = 1 Acute care physiotherapist = 1 Acute care clinical psychologist = 1 Acute care occupational therapist = 1 Stroke consultant = 1 Community physiotherapist = 1 Community speech and language therapist = 1 Community occupational therapist = 1	GP = 2 Nurse = 2	12
Site 06	City, East Midlands; population >300,000	Acute care physiotherapist = 2 Acute care speech and language therapist = 1 Stroke consultant = 1 Community occupational therapist = 1 Community physiotherapist = 1	GP = 1	7

Specialists and generalists reported different levels and quality of communication with each other, influenced by available resources and patients’ care needs. Four themes were identified: 1) generalists and specialists have overlapping roles but are working in silos, 2) referral decision-making processes as influential to generalist-specialist communication, 3) variable quality of communication, and 4) improved dialogue between generalist and specialist services, summarised in Table 2.

**Table 2:** Themes and subthemes.

Theme	Subtheme

Generalists and specialists have overlapping roles but are working in silos	N/A
Referral decision-making process as influential to generalist-specialist communication	Categories of services for (re-)referral
	Criteria for (re-)referral
Variable quality of communication	Barriers to communication
	Enablers to communication
Improved dialogue between generalist and specialist services	N/A

### Generalists and specialists have overlapping roles but are working in silos

Healthcare professionals reported varying degrees of involvement in stroke survivors’ care, depending on a patient’s clinical and social needs. For example, specialists in acute settings acknowledged their role in triaging patients’ rehabilitation needs, making referrals and writing discharge summaries and/or letters. Patients could then be discharged to: ESD (intensive support up to six weeks and stroke reviews, i.e., a structured review of patient health status, medications, cardiovascular events, hospitalisation), Stroke Outreach Service, Stroke Reablement Service, other acute services, or directly to a GP. Some GPs reported having the capacity to offer assessments and referrals to other services. Specifically, some GPs reported referring patients to community neurorehabilitation services in the absence of acute care specialist referral or presence of other identified needs, however, some reported not having an awareness of the specialist services they could refer patients to.

All GPs reported conducting routine reviews of stroke survivors, which include checking patients’ medication, blood pressure, and emotion. Nurses in primary care were also reported (by themselves, GPs and ESD staff) to undertake reviews of stroke survivors’ needs. Time intervals of reviews with generalists were varied, with some reporting conducting the initial review at six weeks, some six months. Participants reported focussing on physical issues and rehabilitation, and empowering patients to access services themselves. However, participants acknowledged that they have different periods of involvement with patients. For example, specialist practitioners in acute settings described their role as intensive and time-limited:

“Site 01

Acute care Speech and Language Therapist (SLT): I think it does certainly vary from area to area […] but the ESD services we refer to have a very time limited period, so they would be withdrawing after six weeks, and then we don’t know what happens […]

Community SLT: We refer them […] But if we’re looking at the pattern actually in terms of when the depression does hit and it does seem to be after six months from the stroke, there are certain peaks and troughs, so actually stopping [involvement] before the six months you’re not actually catching the people before they’ve actually reached that stage.”

Finally, participants reported prioritising patients’ disclosed needs such as rehabilitation (e.g. physiotherapy, speech and language, occupational therapy) and physically visible symptoms, rather than implementing specific clinical guidelines:

“Site 05:

Practice nurse 1: […] we don’t follow this [stroke clinical guideline] sort of in any sort of organised pattern I wouldn’t say

GP 2: So it’s patient-driven at the moment in terms of the symptoms that they volunteer.”

Most emphasised striking a balance between providing comprehensive care with time-limited consultations, by having open discussions about patients’ health and wellbeing, considering the complexity of patients’ post-stroke condition, and conducting/requesting necessary clinical investigations.

### Referral decision-making process as influential to generalist-specialist communication

Generalists and specialists discussed their referral processes for various identified or patient-reported needs to each other and other agencies, which formed two sub-themes: ‘categories of services for (re-)referral’ and ‘criteria for (re-)referral’.

#### Categories of services for (re-)referral

Participants discussed the broad categories they consider when making referrals to either generalist or specialist services: physical and mental health, and social care. Physical health includes medication management, pain, and symptoms of a new stroke. Mental health includes mood (e.g. depression, anxiety), neurocognitive impairments or onset of a mental health crisis. Social care includes daily functioning needs, returning to work, and leisure/social interaction needs.

Generalists reported experiencing difficulties making onward referrals; for instance, not receiving adequate information (e.g. letters, discharge summary, multidisciplinary team meetings) about patients’ needs or health status post-discharge from acute/specialist care:

“Site 02:

Nurse: I think it’s a bit fragmented because of the way things are, you know. […]

GP 2: One small thing I’ve noticed we don’t get, ever get from a hospital a blood pressure reading. It’s really odd.

GP 1: Really?

Acute care nurse: With discharge letter you mean? […]

GP 2: Yeah, never. They [hospital staff] never record blood pressure. Which I thought might be [chorus of yeses] interesting.”

Also, participants acknowledged that stroke survivors’ physical, mental and social care needs interact rather than occur in isolation and can be part of long-term chronic illness or multimorbidity which can be challenging to address:

“Site 04:

Community physiotherapist: We’re quite generic and the problem we have with that is we’ve had patients referred on who the main cause, […] instigator of mood disorder is communication. And so you’ve got profoundly aphasic patients where your non-specialist counselling’s very, well it can be counterproductive and that’s we’ve kind of had to pull people back from that because it’s actually causing real problems.”

Other challenges include patients having a stroke outside their local geographical area, limited capacity or availability of services, and the inability of some healthcare professionals to make direct referrals for identified physical, mental or social care needs. For example, speech and language therapists highlighted that some services require stroke survivors to self-refer, a barrier to those with communication issues:

“Site 01:

Acute SLT: And how complex that system [Single Point of Access – SPA] would be if you were a stroke patient trying to refer yourself.

Acute occupational therapist (OT): Yes.

Acute SLT: And particularly a stroke patient trying to re-refer yourself who’s got a communication problem.

Acute OT: Yes, exactly.”

#### Criteria for (re-)referral

Referral decision-making processes involve various healthcare professionals at all stages of post-stroke care. Decisions to refer back to either specialists or generalists are influenced by practitioners’ discussion of patients’ needs in consultations and during the course of treatment/rehabilitation, taking into consideration expressed needs which includes severity and persistence of symptoms, urgency, issues in the background of a patient’s life after having a stroke, and any improvements or positive changes poststroke. One GP described the criteria they would consider before making a referral during the clinical vignette exercise in the focus group discussion:

“I don’t think I would necessarily be looking at a referral back immediately for this patient, I’d probably want to just see over a number of consultations what the problem was, whether there were any background issues (GP, Site 06).”

While some healthcare professionals (e.g. acute care physiotherapists, stroke consultants) make direct referrals to services (e.g., ophthalmology, outpatient therapy, GP), others (e.g., psychologists, community physiotherapists/OTs) liaise with GPs who then make referrals on their behalf. Occasionally, stroke survivors can self-refer to services (e.g. physiotherapy, ESD team), as encouraged by healthcare professionals; however, issues concerning this were identified by SLTs (see Categories of services for (re-)referral).

Moreover, generalists and specialists reported linking patients to third sector services and offering carers information and advice. The availability of services, and knowledge of these influence referrals: “One of the other things to consider is […] are all of these services actually available in this area? (GP, Site 04)”. Participants reported limited guidance on referral to services other than health and social care (e.g. education), leading to uncertainty about where to signpost patients.

For many specialists, “the GP practice is a constant” (Community OT, Site 05) in patients’ care, providing long-term medical management, and supporting healthcare professionals who are less familiar with a patient’s medical history/needs:

“Site 04:

Acute care nurse: Yeah. If we’ve got any queries then we would ring before discharge […]

Acute care physiotherapist: […] GPs are often our medical back up if I can’t get a question answered or it’s a new thing

GP 1: Yeah, yeah generally people leave messages…”

Finally, for generalists, findings from reviews and/or home visits, Quality and Outcomes Framework (QOF) indicators (i.e., performance management and payment system for UK primary care) such as blood pressure, secondary prevention and related conditions including diabetes guide referral decision-making:

“Site 05:

Practice nurse 2: […] we would do a lot of blood pressure, weight, annual bloods and that sort of thing. Depression, we always ask about that anyway. Lifestyle, um…

GP 2: Stuff driven by QOF points.

Practice nurse 2: Basically, yeah.”

### Variable quality of communication

Generalist-specialist communication was characterised by variable frequency (e.g., regular, ad hoc) and methods (e.g., letters, phone calls, face-to-face meetings). This combination of formal and informal communication structures was judged by study participants as of inconsistent quality. For example, discharge summaries may come with limited information:

“Site 04:

Community physiotherapist: […] we do try and communicate as much as we can with our acute care colleagues […] to see if there are any up-and-coming discharges or we might be talking to them about the referrals that they normally send to us, we talk about where they are with that discharge and how the patient’s doing […]

Acute OT: […] from the acute side once they’ve left the hospital they are handed over to the community teams. I think communication-wise obviously there will be a discharge summary that would be sent to the GPs and that would be done largely by junior doctors. I suppose may not contain any information about therapy in it so I suspect there might be a lack of information around a patient’s function that goes to you guys?

GP 1: Yeah. I mean that’s one of the things that we do struggle with sometimes is, you know, we’ll get a nice discharge […] But the more tricky ones we don’t often get sort of the information that we can necessarily act on.”

Two sub-themes were identified, ‘barriers to communication’ and ‘enablers to communication’, which we discuss in turn.

#### Barriers to communication

Having different information technology (IT) systems was problematic, delaying important information exchange: “[…] everybody’s on a different system as well. […] Even within our Early Supported Discharge team I’m on SystmOne but the rest of the team aren’t (Specialist SLT, Site 05)”. Challenges met with the unavailability of shared formal IT systems for information exchange was compounded by not having a shared language – that is, using different abbreviations or assessment tools – between generalists and specialists resulting in inaccurate and/or incomplete information being shared. Some specialists also reported that other professionals were unfamiliar with their services: “lots of GPs don’t actually know that we [community neurological rehabilitation] exist, or that they can refer back to us’ (Community occupational therapist, Site 01)”. Generalists reported limited knowledge of different healthcare professional role boundaries:

“Site 05:

GP 2: As a group of therapists who do you, can you take referrals from? Is it just general practitioners or other therapists?

Acute physiotherapist: From neuro outpatients […] we got them directly from the therapists at the acute side and then we also got them from GPs. […]

Acute occupational therapist: Does that mean that they [referrals] can’t come from for example a nurse in a GP practice?

Acute physiotherapist: I don’t think so […]

GP 2: As you just said I don’t know who to refer to…”

Finally, changes in commissioned services were influential to practitioners’ ability to communicate with each other: “there is no stability in what’s commissioned and who commissions it and what the agendas are around commissioning […] we don’t get an opportunity to know who to hang on to (Specialist physiotherapist, Site 04).”

#### Enablers to communication

Multidisciplinary teams (MDTs) in community-based services were considered vital to planning post-discharge care, making referrals, and obtaining comprehensive accounts of stroke survivors’ post-stroke conditions, especially for those with complex needs:

“[…] when they [hospital] discharge a patient they usually do discharge the patient with a care package and so occupational are involved, there is that continuing care and it’s just a matter of them being picked up by us in MDT in the MDT settings. (GP, Site 02).”

Familiarity and established interprofessional relationships were valued for informal information sharing and communication practices: “I know [Staff Name] well from working on the unit and, you know, so it helps when you know people [chorus of yeses] hugely (Specialist physician, Site 05)”. Participants appreciated having specific liaison staff who facilitate interprofessional communication. Finally, having appropriate communication tools including dedicated telephone numbers, hospital bleeps, written letters and shared information systems enabled information handover, linkage with other services, and long-term care management: “if we’ve got a query […] we’ll just ring up […] also if we One Call [NB: a dedicated telephone number for referring anybody to community services] it and for some reason the fax with the detailed information goes astray (Specialist physiotherapist, Site 03)”.

### Improved dialogue between generalist and specialist services

Study participants exchanged ideas for improving generalist-specialist communication. A stroke discharge management plan or summary outlining patients’ information (e.g. tests undertaken, relevant findings), through a query or checklist, was suggested by generalists and specialists. Some highlighted that templates for recording patients’ information should be clear and shared across services rather than duplicated:

“[…] you want to know what the diagnosis is and you want to know what has been done, what results of tests were, and you don’t want to just know GP checked BP because it was high, how high was it and what you did about it, what was on the discharge (GP, Site 03).”

Moreover, participants reported valuing interactions with primary and secondary care colleagues to discuss the patients under their care, thereby bridging the gap between providers. Specifically, the ability to directly contact other healthcare professionals (e.g. by telephone, email) on an ad hoc or informal basis:

“Site 03:

GP: The neurologist […] if you email him he’ll usually fire you back an email and if you put plenty of detail in it he’ll usually give you a good reply […] the more you put in the better you get back […]

Acute care physiotherapist: So that people normally ring up the stroke secretary and leave a message, and then she badgers the consultants till they ring somebody back generally [all laugh].”

Finally, many participants reported that shared information systems and up-to-date healthcare professional contact details enhanced access to patient information and improved care: “the biggest benefit is that we can see what you’re doing as well as you all can see what we’re doing […] it’s so much easier now when we see the messages (GP, Site 04).”

## Discussion

### Summary

Generalists and specialists acknowledge their overlapping roles in providing information and support to patients and each other. Referral decision-making is informed by clinical expertise, interactions with colleagues, knowledge and availability of relevant services, and discussions with patients rather than formulaic adherence to clinical guidelines. Effective interprofessional communication is characterised by being able to contact colleagues and having adequate resources. Generalist-specialist communication can be irregular and is affected by system and organisational issues, such as changes in service commissioning, differences in language, and resource constraints, which hamper adequate information sharing. Healthcare professionals valued interprofessional communication, but recognised the need for improvement initiatives.

### Comparison with existing literature

Few studies have explored UK generalist and specialist care providers’ communication processes concerning stroke care, and barriers and enablers to this. Such studies typically explore patients’ and carers’ perspectives [14,16] and occasionally, healthcare professionals’ [4,26,27].

Consistent with this study, previous research shows that understanding and appreciating professional roles and function is a key skill and ‘support and value’ mechanism underpinning interprofessional collaboration [28,29,30]. Specifically, GP surgeries were considered by some participants as longstanding support systems for managing stroke [14,20,31]. Whereas, specialists (e.g. therapists) described their roles as having a fixed end, and as such are temporally bounded [21]. Therefore, role boundaries need to be agreed by those in the care pathway to ensure expectations are managed.

Patients experience unmet long-term needs after stroke that could potentially be addressed in general practice [3,4,32]. However, generalists reported relying on stroke survivors to disclose their needs, despite some stroke survivors (e.g. those in care homes) being more passive actors in the management of their care [15].

Moreover, our findings suggest varied levels of awareness of roles and service functions, which hinders interprofessional communication. This variation in knowledge across generalists and specialists indicates limited organisational infrastructure or support for joint communication [33], which could be a reflection of the differences in how these healthcare professionals and services are financed in England [34]. An understanding of care pathways, team members’ roles, and areas of overlap is important for facilitating timely information exchange, collaborative working, development of rehabilitation plans and provision of long-term care [4,32]. Thus, complete and consistent knowledge of roles and services amongst generalists and specialists is needed.

Current UK stroke guidelines encourage interprofessional collaboration, so patients are referred to appropriate services in a timely fashion [2]. Adherence to clinical guidelines could benefit patient care and outcomes [35]; yet, a systematic review of healthcare professionals’ adherence to stroke-specific guidelines showed inconsistent adherence, dependent on the generality or specificity of guidelines [36]. Our data show that generalists and specialists have limited time for interprofessional communication and are faced with the challenge of not having shared language or terminology with each other, thereby reducing their capacity to communicate [21].

Despite the knowledge that well-coordinated transitions between stroke care phases are important [32,37] the study participants reported encountering obstacles to implementing guidelines into practice such as limited involvement in a patient’s care derived from a need to discharge from one care environment to another, and the absence of fluid information transfer due to changes in team or service structures. In line with previous research, our findings suggest that efforts to enhance adherence to stroke guidelines in primary-secondary care settings are needed to overcome inconsistencies in care provision [32,33]. Additionally, policy makers should be aware of the complex care pathways which healthcare professionals navigate and take these into account when making decisions concerning integrated care.

Effective communication is essential for interprofessional collaboration [29,38]. Technology (e.g. email) can enhance primary-secondary care communication [39]. However, in this study, these tools appear to be inconsistently available across providers and geographical areas. Whilst several modes of contact were identified, as previously found [19,40,41], participants recommended shared information systems as a way to bridge the communication gap between specialists and generalists. There is some evidence that shared information systems can improve both information sharing between healthcare professionals [9,35,36] and care co-ordination including in stroke [32,42]. Standardised templates or assessment tools for recording patient information can also be useful for facilitating communication between different providers [40,41,43,44]. However, study participants emphasised that these should be developed such that different providers mutually understand how to use these so that high-quality information is shared. Indeed, establishing good interprofessional relationships that supports freely accessible and open communication was valued by all study participants.

### Strengths and limitations

To our knowledge, this is the first study exploring stroke-related interprofessional communication between generalists and specialists. Although we had a large sample of 48 healthcare professionals, only six focus groups were conducted. The study was limited to two geographical areas in England. Nevertheless, robust qualitative methods were used, such as adding group-based exercises based on external sources and fictional scenarios to the focus group questions, capturing a range of divergent and complementary views [45].

Participants volunteered to participate in this study and may therefore be better informed on this topic than non-participants who may be uninvolved in stroke-related communication between generalists and specialists. Nevertheless, participant accounts were consistently reported, and echo existing research on interprofessional communication, and interprofessional collaboration for long-term stroke care. Therefore, the issues detailed are likely to be relevant and transferrable to similar contexts and healthcare systems.

### Implications for research and practice

Gaps in communication between generalists and specialists need to be addressed to enable better support of patients after stroke. Suggested improvements include the presence of opportunities to develop understanding of professional roles, and flexibility to communicate with each other, including using informal channels [11,12,23]. The development of an effective integrated care service is a long-term process [33]. While these modifications have face validity, there is an absence of empirical evidence of their impact, so implementation should be accompanied by evaluation. Enabling interprofessional communication through technology is not a panacea for the fragmentation between generalists and specialists.

## Conclusion

Generalist and specialist healthcare professionals recognise the need for better communication with each other. Pragmatic methods to improve this are available, but current care continues to be characterised by silo-based working which ignores the important contribution that each sector can make. Healthcare professionals need to be involved when organisations develop agreements or commitments to implement integrated care to ensure integrated care elements (e.g., local referral pathways, communication tools) valued by practitioners are included. Failure to bridge this communication gap will result in people with stroke continuing to experience unmet stroke needs and fragmented care.
